# Supplementary material for: Demographic Analysis of Shortfin Mako Shark (Isurus oxyrinchus) in the South Pacific Ocean
Source: Animals (Basel). 2022 Nov 21;12(22):3229. doi: 10.3390/ani12223229 (PMC9687030; doi:10.3390/ani12223229)
Supplement: Supplementary file 1 [file animals-12-03229-s001.zip › animals-1957107-supplementary.pdf]

Table S1. M_s (year⁻¹) estimated using 14 indirect approaches.

ID	Method	Relies on parameters	Male M_s (yr⁻¹)	Female M_s (yr⁻¹)
M1	Campana et al. [64]	a_{max}	0.148	0.102
M2	Hoeing [65]	a_{max}	0.176	0.114
M3	Then et al. [66]	a_{max}	0.246	0.166
M4	Then et al. [66]	a_{max}	0.206	0.133
M5	Hamel [67]	a_{max}	0.167	0.109
M6	Zhang and Megrey [68]	a_{max} *and* k	0.190	0.104
M7	Jensen [69]	a_{mat}	0.197	0.083
M8	Hisano et al. [34]	a_{mat} *and* t_0	0.180	0.094
M9	Frisk et al. [70]	a_{mat}	0.134	0.069
M10	Cubillos et al. [71]	t_0 *and* k	0.135	0.116
M11	Jensen [69]	k	0.139	0.122
M12	Frisk et al. [70]	k	0.156	0.148
M13	Hamel [67]	k	0.152	0.134
M14	Then et al. [66]	k *and* L_∞	0.106	0.093
		Median	0.167	0.113

Table S2. Estimated demographics in various scenarios based on a 2-year reproductive cycle.

2-year reproductive cycle							
**Case**	**Sex**	**λ (year⁻¹)**	**Lower CL**	**Upper CL**	**r (year⁻¹)**	**Lower CL**	**Upper CL**
Scenario A (F = 0)	Combined	1.098	1.036	1.165	0.093	0.036	0.153
	Male	1.143	1.050	1.243	0.133	0.049	0.217
	Female	1.045	0.978	1.103	0.044	-0.022	0.098
Scenario B (F = 0.8)	Combined	0.949	0.875	1.025	-0.053	-0.133	0.024
	Male	0.977	0.859	1.089	-0.025	-0.152	0.086
	Female	0.911	0.822	0.988	-0.094	-0.196	-0.012
Scenario C (F = 0.6)	Combined	0.983	0.910	1.058	-0.017	-0.094	0.056
	Male	1.017	0.904	1.127	0.015	-0.101	0.120
	Female	0.942	0.858	1.016	-0.061	-0.154	0.016
Scenario D (F = 0.4)	Combined	1.020	0.952	1.092	0.019	-0.049	0.088
	Male	1.058	0.951	1.164	0.055	-0.051	0.152
	Female	0.975	0.894	1.044	-0.026	-0.112	0.043
Scenario E (F = 0.2)	Combined	1.058	0.993	1.129	0.056	-0.007	0.121
	Male	1.100	1.001	1.204	0.094	0.001	1.186
	Female	1.010	0.935	1.072	0.009	-0.067	0.070
Scenario P1 ($F_{immature}$ = 0)	Combined	1.050	0.986	1.118	0.048	-0.015	0.111
	Male	1.090	0.993	1.192	0.085	-0.007	1.175
	Female	1.005	0.932	1.068	0.004	-0.070	0.066
Scenario P2 (F_{mature} = 0)	Combined	1.000	0.933	1.073	-0.001	-0.069	0.071
	Male	1.032	0.926	1.143	0.030	-0.077	0.133
	Female	0.960	0.890	1.027	-0.042	-0.116	0.026
Scenario P3	Combined	1.026	0.951	1.102	0.025	-0.050	0.097

($F_{immature\ male}$ = 0)	Male	1.091	0.994	1.192	0.086	-0.007	0.176
	Female	0.917	0.827	0.997	-0.088	-0.190	-0.003
Scenario P4	Combined	0.989	0.914	1.068	-0.012	-0.090	0.065
($F_{mature\ male}$ = 0)	Male	1.033	0.926	1.141	0.031	-0.077	0.132
	Female	0.913	0.825	0.990	-0.092	-0.192	-0.011
Scenario P5	Combined	0.997	0.934	1.056	-0.004	-0.068	0.055
($F_{immature\ female}$ = 0)	Male	0.979	0.862	1.091	-0.023	-0.148	0.087
	Female	0.999	0.928	1.061	-0.002	-0.074	0.059
Scenario P6	Combined	0.972	0.912	1.037	-0.029	-0.093	0.036
($F_{mature\ female}$ = 0)	Male	0.978	0.863	1.089	-0.024	-0.147	0.085
	Female	0.960	0.839	1.026	-0.041	-0.114	0.026

Table S3. Estimated demographics in various scenarios based on a 3-year reproductive cycle.

3-year reproductive cycle							
**Case**	**Sex**	**λ (year^{-1})**	**Lower CL**	**Upper CL**	**r (year^{-1})**	**Lower CL**	**Upper CL**
Scenario A	Combined	1.063	1.008	1.119	0.061	0.008	0.113
(F = 0)	Male	1.097	1.015	1.183	0.092	0.015	0.168
	Female	1.026	0.962	1.080	0.025	-0.039	0.077
Scenario B	Combined	0.925	0.860	0.993	-0.079	-0.151	-0.008
(F = 0.8)	Male	0.942	0.833	1.045	-0.062	-0.183	0.044
	Female	0.900	0.818	0.973	-0.106	-0.201	-0.028
Scenario C	Combined	0.957	0.892	1.021	-0.045	-0.114	0.021
(F = 0.6)	Male	0.979	0.875	1.075	-0.023	-0.134	0.073
	Female	0.929	0.849	0.998	-0.075	-0.164	-0.002
Scenario D	Combined	0.991	0.929	1.054	-0.010	-0.074	0.052
(F = 0.4)	Male	1.017	0.923	1.112	0.016	-0.081	0.106
	Female	0.959	0.882	1.025	-0.042	-0.125	0.024
Scenario E	Combined	1.025	0.968	1.084	0.024	-0.032	0.081
(F = 0.2)	Male	1.055	0.966	1.144	0.053	-0.034	0.135
	Female	0.991	0.923	1.050	-0.009	-0.080	0.049
Scenario P1	Combined	1.017	0.961	1.076	0.017	-0.040	0.074
($F_{immature}$ = 0)	Male	1.045	0.959	1.136	0.043	-0.042	0.128
	Female	0.987	0.919	1.046	-0.013	-0.085	0.045
Scenario P2	Combined	0.975	0.919	1.036	-0.026	-0.084	0.036
(F_{mature} = 0)	Male	0.998	0.903	1.091	-0.003	-0.102	0.087
	Female	0.947	0.882	1.008	-0.055	-0.126	0.008
Scenario P3	Combined	0.990	0.921	1.058	-0.011	-0.082	0.056
($F_{immature\ male}$ = 0)	Male	1.045	0.956	1.134	0.044	-0.045	0.126
	Female	0.904	0.821	0.980	-0.102	-0.197	-0.020
Scenario P4	Combined	0.962	0.898	1.031	-0.040	-0.107	0.030
Case 20 ($F_{mature\ male}$ = 0)	Male	0.998	0.907	1.093	-0.003	-0.098	0.089
	Female	0.899	0.816	0.971	-0.107	-0.023	-0.029
Scenario P5	Combined	0.973	0.916	1.026	-0.027	-0.088	0.026
Case 21 ($F_{immature\ female}$ = 0)	Male	0.942	0.833	1.040	-0.062	-0.183	0.040
	Female	0.982	0.913	1.039	-0.019	-0.091	0.039

Scenario P6<br>Case 22 ($F_{mature\ female}$ = 0)	Combined	0.951	0.895	1.008	-0.050	-0.111	0.008
	Male	0.942	0.835	1.042	-0.061	-0.180	0.042
	Female	0.948	0.882	1.009	-0.054	-0.126	0.009

Table S4. A_t for the 2- and 3-year reproductive cycles under unfished conditions.

2-year reproductive cycle

$$A_t = \begin{bmatrix} 0 & 0 & 0 & 2.719 & 0 & 0 & 0 & 5.123 \\ 0.435 & 0.729 & 0 & 0 & 0 & 0 & 0 & 0 \\ 0 & 0.119 & 0.439 & 0 & 0 & 0 & 0 & 0 \\ 0 & 0 & 0.409 & 0.838 & 0 & 0 & 0 & 0 \\ 0.435 & 0 & 0 & 0 & 0.871 & 0 & 0 & 0 \\ 0 & 0 & 0 & 0 & 0.022 & 0.603 & 0 & 0 \\ 0 & 0 & 0 & 0 & 0 & 0.290 & 0 & 0.893 \\ 0 & 0 & 0 & 0 & 0 & 0 & 0.893 & 0 \end{bmatrix}$$

3-year reproductive cycle

$$A_t = \begin{bmatrix} 0 & 0 & 0 & 1.813 & 0 & 0 & 0 & 5.122 & 0 \\ 0.435 & 0.729 & 0 & 0 & 0 & 0 & 0 & 0 & 0 \\ 0 & 0.119 & 0.439 & 0 & 0 & 0 & 0 & 0 & 0 \\ 0 & 0 & 0.408 & 0.838 & 0 & 0 & 0 & 0 & 0 \\ 0.435 & 0 & 0 & 0 & 0.872 & 0 & 0 & 0 & 0 \\ 0 & 0 & 0 & 0 & 0.022 & 0.603 & 0 & 0 & 0 \\ 0 & 0 & 0 & 0 & 0 & 0.290 & 0 & 0 & 0.893 \\ 0 & 0 & 0 & 0 & 0 & 0 & 0.893 & 0 & 0 \\ 0 & 0 & 0 & 0 & 0 & 0 & 0 & 0.893 & 0 \end{bmatrix}$$
